# Barriers to gender equity for female healthcare academicians and researchers in Pakistan: Is it still an issue?

**DOI:** 10.3389/fpubh.2022.981178

**Published:** 2023-01-04

**Authors:** Madeeha Malik, Azhar Hussain, Ayisha Hashmi, Hamza Saeed, Hafsa Azhar, Aqsa Sajjid Abbasi

**Affiliations:** ^1^Department of Pharmacy Practice, Hamdard Institute of Pharmaceutical Sciences, Hamdard University Islamabad, Islamabad, Pakistan; ^2^Cyntax Health Projects, Pvt., Ltd., Contract Research Organization and Corporate Firm, Islamabad, Pakistan; ^3^Institute of Applied Sciences and Technology, Haripur, Pakistan

**Keywords:** academician, gender equity, Pakistan, female, healthcare professionals

## Abstract

**Introduction:**

Gender inequity in healthcare academia is rising. Female healthcare professionals are struggling to balance their work and family leading to reduced productivity and disparity in three main areas of academic evaluation including teaching, service and research.

**Objective:**

The objective of the current study was to explore perceptions of healthcare academicians regarding gender equity and its associated barriers in healthcare academia in Pakistan.

**Methodology:**

A qualitative study design was used. Study site for this research included medical colleges, pharmacy schools and healthcare educational institutes. Study respondents included healthcare professionals including doctors and pharmacists working as faculty members. Snow ball sampling was used and interviews were conducted till saturation point was achieved. All interviews recorded were transcribed verbatim. Transcribed interviews were then subject to thematic analysis and were analyzed for relevant content.

**Results:**

Thematic analysis of the study yielded many themes and sub themes. The main themes include: Gender equity an issue for healthcare academicians; Barriers toward promoting gender equity among healthcare academicians; Perceived teaching effectiveness among female healthcare academicians; Professional relationship of female healthcare academicians; Representation of female healthcare academicians at leadership positions; Research opportunities for female healthcare academicians; impact of academia as profession on married life and Strategies for improving gender equity disparities in academia of healthcare profession. The results showed that almost all the females as well as male healthcare academicians were of the view that female academicians are competent, hardworking and committed with their jobs. Mixed responses were observed regarding teaching effectiveness of female academicians. Half of the females as well male healthcare academicians thought that female were more effective teachers while other half was of the view that teaching effectiveness is based on individual trait irrespective of gender.

**Conclusion:**

The results of the present study concluded that majority of the male as well as female healthcare professionals perceived gender equity as an issue in academia in Pakistan, especially in underprivileged areas. Non-conducive work environment, harassment at workplace and cultural issues were the main barriers identified toward gender equity in healthcare academia in Pakistan.

## Introduction

Equity is defined as “distribution of variable number and types of resources within a group to achieve results in equal magnitude”. However, in contrast, the phenomenon of equality follows “one size fits all” approach which promotes distribution of equal resources among individuals (1). Gender disparities in the field of healthcare has been subject of interest from many years and increasing trends of gender inequity in healthcare academia has been witnessed over the last decade (2). Despite rigorous efforts, the disparity lies in availability of growth opportunities and resources as well as rewards for both genders. It has been observed that women are subjected to both inequity and inequality in the field of academia by assigning them greater work load in terms of teaching and advisory duties which further decreases their participation in research as compared to their male colleagues (3, 4). Throughout the field of healthcare academia, managerial positions are mostly led by males allowing them to take decisions as well as have more opportunities for social networking. The recent global pandemic has further increased the gender gap (5). Female healthcare professionals are striving hard to balance their work and family commitments leading to reduced productivity and disparity in three main parameters of academic evaluation including teaching, professional service and research. In the recent years, the focus on improving women's representation in the fields of science, technology, engineering, and mathematics (STEM) has increased; however, interventions focused on increasing gender equity in these fields still has long way to go (6). These gender disparities are more prominent within the field of academia. The proportion of women faculty in STEM remains low despite of the increased women acquiring their post graduate degrees in the past few years (7). Numerous factors contribute toward under representation and disparity including workplace stereotypes, low social support from colleagues and uncomfortable work environments. Harassment and discrimination are also common factors contributing to the rising gender inequity. Inadequate opportunities for mentoring, networking and professional growth also increases the gender gap in academia (8, 9).

Among all the countries, gender disparity is common in South Asia including Pakistan (10). According to World Economic Forum, Pakistan is on number 56th among the 58 countries that have progressed toward improving status of gender equity in the country despite of the fact that gender equality has already been a part of the country legislation since 1973 (11). In Pakistan, women are expected to be responsible for taking care of households while decision making is considered to be the responsibility of male counterparts (12). Literature review has highlighted that female faculty members teaching in universities in Pakistan had experienced gender discrimination and had low levels of job satisfaction. Female academicians are usually not part of committees involved in decision making and only few females have access to leadership positions in academia. The chances for professional growth in academia is low for female academicians in the country as well as they have to face more discriminatory barriers in the field (13, 14). There is also lack of equal access to professional development opportunities. Limited research has been conducted to identify barriers linked to gender equity in healthcare academia. Therefore, the present study was designed to explore the perceptions of healthcare professionals regarding gender equity in healthcare academia in Pakistan.

## Methodology

### Study design

A qualitative study design was used to explore perceptions of healthcare professionals toward gender equity in healthcare academia in Pakistan.

### Study site and respondents

Study site for this research included medical colleges, pharmacy schools and healthcare educational institutes. Study respondents included healthcare professionals including doctors and pharmacists working as faculty members.

### Ethical approval

Ethical approval was obtained for this study by the Ethical Review Board of Hamdard University, Islamabad, Pakistan (ERB/HUIC/295). A comprehensive description of the study was introduced to the participants at the first of the interview; they were allowed to reject answering or withdrawing at any time thereafter. The participants were assured that their answers would be kept confidential and that their names would not be disclosed during the study and in the final report.

### Sampling technique and sample size

Non probability sampling technique was used i.e., Snow ball sampling was adopted as it is the best way of identifying the respondents having common characteristics, experience and job profile which were difficult to contact. Any pharmacist and doctor of both genders teaching in any public or private university/medical college with at least 1 year experience willing to participate was included in the study. Interviews were conducted till saturation point was achieved. The sample size at saturation point for different respondents was: Female Pharmacists (*n* = 9), Female Doctors (*n* = 10), Male Pharmacists (*n* = 10) and Male Doctors (*n* = 11).

### Study tool

After extensive and critical literature review a semi structured interview guide was developed and used as a study tool (3, 4, 9, 13, 15). To get detailed views of respondents, the focus was to design questions as open as possible. The tool comprised of 3 major sections with a total of 15 questions. First section included questions regarding gender equity as an issue and related barriers toward promoting gender equity for healthcare academicians. The second section was comprised of questions about perceived traits, competency, skill set, teaching effectiveness and professional relationship with male colleagues. The third section consisted of questions regarding representation at leadership positions, research opportunities, impact of academia as profession on eligibility for marriage and suggestions for promoting gender equity in Pakistan. Face and content validation of interview guide was done by panel of experts.

### Interview conduction

The selected respondents were contacted either personally or through phone for getting interview appointment date and time. Written consent was obtained from the study respondents before conducting interview. When necessary, probing questions were used. Each interview lasted approximately 20–30 min. Every respondent was given chance to express his/her views at the end of interview session. All interviews were conducted in the local language i.e., Urdu. Interviews were conducted till point of saturation was achieved. All interviews were recorded after getting permission from the respondents.

### Thematic analysis

All interviews recorded were transcribed verbatim. The interviews were transcribed verbatim and were then subject to thematic analysis and were drawn using bottom up approach.

## Results

### Demographic characteristics of the respondents

Majority of the respondents from both groups i.e., male and female pharmacists as well as doctors were in the age group of 36–45 years. Most of the respondents (72.5%; *n* = 29) from both groups were having postgraduate degree. Most of them (32.5%; *n* = 13), were having experience of 6–10 years (Table 1).

**Table 1:** Demographic characteristics of the respondents.

		**Pharmacists**		**Doctors**	
		**Females (*N =* 09) *n* (%)**	**Males (*N =* 10) *n* (%)**	**Females (*N =* 10) *n* (%)**	**Males (*N =* 11) *n* (%)**
Sector	Public	4 (44.5 %)	5 (50 %)	6 (60 %)	5 (45.5 %)
	Private	5 (55.5 %)	5 (50 %)	4 (40 %)	6 (54.5 %)
Qualification	Bachelors	2 (22.2 %)	3 (30 %)	4 (40 %)	2 (18.2 %)
	Postgraduate	7 (77.8 %)	7 (70 %)	6 (60 %)	9 (81.8 %)
Age	25–35 years	2 (22.2 %)	3 (30 %)	2 (20 %)	2 (18.2 %)
	36–45 years	4 (44.5 %)	5 (50 %)	3 (30 %)	5 (45.5 %)
	Greater than 45 years	3 (33.3 %)	2 (20 %)	5 (50 %)	4 (36.3 %)
Teaching experience	1–2 years	2 (22.2 %)	3 (30 %)	2 (20 %)	2 (18.2 %)
	3–5 years	2 (22.2 %)	2 (20 %)	3 (30 %)	3 (27.3 %)
	6–10 years	3 (33.4 %)	3 (30 %)	3 (30 %)	4 (36.3 %)
	Greater than 10 years	2 (22.2 %)	2 (20 %)	2 (20 %)	2 (18.2 %)

### Theme 1: Gender equity an issue for healthcare academicians

Most of the female healthcare professionals were of the view that gender equity is not an issue for female healthcare professionals in academia in Pakistan. However, most of them stated that it could be seen as a major barrier for females working in other fields and underprivileged areas. On the other hand, it was interesting to notice that majority male healthcare professionals perceived gender equity as an issue in Pakistan.

“*Gender equity is not an issue of Pakistan especially for female pharmacists working in academia. In every province female faculty is present beyond the equity issue. However, in public sector universities, females might be uncomfortable with their environment and not working but vacancies are available for them”. (F.Pharm.01)*

“*According to me gender equity is not a big issue for female doctors. As females have progressed over the years and are giving tough competition to the male doctors in academia, research as well as practice in Pakistan. I think it all depends on one's personal will and skills”. (F.Doc.03)*

“*Yes, I think gender equity is not only an issue for academician but is a major challenge seen for overall employment opportunities. I think in Pakistan, in recruitment or selection process for almost every employment opportunity, male dominance and preference exists and I think male candidates are preferred till date over female candidates. In my opinion, this practice equally exists in both public and private sector”. (M.Pharm.03)*

“*Yes, gender equity is an issue in Pakistan. Although, a good number of female doctors are working as faculty in various medical colleges but usually they are not offered administrative positions at times due to their family commitments irrespective of their professionalism”. (M.Doc.01)*

### Theme 2: Employment to population ratio with respect to female academician in healthcare profession

Mix responses were seen among the respondents. Female pharmacists were of the view that employment to population ratio of female academician was low while female doctors thought that their employment ratio was sufficient. On the other hand, nearly half of the male pharmacists thought that employment ratio was low while the other half were of the view that it was appropriate. In contrary, majority of the male doctors were of the view that employment to population ratio with respect to female doctors was good.

“*I think there are more female doctors working in academia as it is a much relaxing job and it is easy for the females to manage work and family life”. (F.Doc.02)*

“*No, I think less females are employed with respect to population in academia due to different issues including transportation, work-life balance, and different family pressures”. (F.Pharm.04)*

“*Obviously not, because if you look at the population ratio, more than 50% of the population is female but if you look at the employment opportunities, or if you conduct a general survey in academics, you will find an overwhelming majority of males working in universities as compared to female employs. Therefore, I think population ratio is not justified at all and the requirement of female academician is not fulfilled at all”. (M.Pharm.07)*

“*I think there are more females working in academia than males. I think this trend is similar in both sectors but more evident in private sector at present, due to less working hours and shifts”. (M.Pharm.02)*

“*I believe appropriate number of females doctors are working as academicians. This is due to less job stress and work load in teaching as compared to practice”. (M.Doc.03)*

### Theme 3: Barriers toward promoting gender equity among healthcare academicians

Different barriers identified by both male and female healthcare professionals toward promoting gender equity among healthcare academicians included mainly as lack of support at workplace, workplace harassment and cultural issues.

#### Support at workplace

Majority of the female as well as male healthcare professionals were of the view that lack of support at work place is one of the biggest challenges faced by the female healthcare academicians due to which they are not able to balance between work and family.

“*I think lack of support at workplace in terms of provision of transport & day care facilities and struggle to prove oneself in a highly male dominance society are one of the important reasons towards lack of gender equity opportunities in this society” (F.Pharm.03)*

“*Family commitments are a problem for a female as her job is usually considered as a secondary thing. Most of the employers do not provide transportation and child daycare facilities which is one of the biggest issues for the females to manage their job with family life”. (F. Doc.06)*

“*I think lack of supportive work and family environment is one of the barriers which is most commonly faced by the female academicians” (M.Pharm.02)*

“*In my opinion, most of the organizations do not provide transport and daycare facilities due to which most of the females fail to manage their work life balance and often quit” (M.Doc.04)*

#### Workplace harassment

Most of the female as well male healthcare professionals considered workplace harassment as a barrier toward gender equity in Pakistan.

“*Yes, unfortunately female academicians have to face this issue. This happens relatively more in public sector. However, I have also seen some females taking undue advantage and when they don't get this they blame a male for harassment” (F.Pharm.01)*

“*Yes, I think workplace harassment is one of the major issues faced by female academicians. There is more harassment in public setup as people come from different backgrounds and possess different mindset, especially lower rank people don't know how to behave with females. However, in private setup this is less because departments are more interconnected due to which there are less harassments issues” (F.Doc.06)*

“*Well, in my opinions the topmost barrier is lack of suitable work environment and workplace harassment” (M.Pharm.02)*

“*I think workplace harassment is one of the issues faced by female academicians especially more commonly in universities located in underprivileged areas of Pakistan” (M.Doc.07)*

#### Salary disparity

Low salary was identified as one of the issues faced by both genders. Majority of the female as well as male respondents stated that usually the salary in academia is set with respect to qualification, research publication and experience and the market trends follow this for both genders.

“*I do not see any disparity in salary of female academicians versus males as the salary bracket is set according to qualification, research profile and experience. However, overall we are underpaid in academia” (F.Pharm.05)*

“*Salary in academia is according to qualification and experience and does not depend upon gender. However, I think academicians are low paid in the country” (F.Doc.02)*

“*I think salary in academics follow market trends set as per qualification and experience equally for both genders” (M.Pharm.01)*

“*I do not find any disparity in the salary of female or male academicians as it is defined by a criterion irrespective of gender. But I must admit that academicians are paid quite less” (M.Doc.02)*

#### Emotional intelligence

Emotional Intelligence was identified as one of the issues faced by females. Almost all the female as well as male respondents stated that emotional instability is one of the challenges faced by female academicians.

“*Yes, it is one of the major issue faced by females as if you can't understand your emotions you will stick to them missing rationality” (F.Pharm.01)*

“*Yes this is an issue for the females as most of them don't come relaxed from home so all the stress is reflected at workplace as they can't manage their emotions” (F.Doc.07)*

“*Yes, emotional instability is an issue faced by female academicians. Main reason are lack of decision power and at times getting influenced by environment” (M.Pharm.08)*

“*Yes, definitely, females have this issue, they lack patience and easily get influenced by whatever they see or listen. Females rely a lot on others and quickly build their perceptions without getting into depth of scenario” (M.Doc.02)*

#### Cultural issues

All the male and female academicians agreed that culture was an important barrier toward gender equity especially in case of remote areas including KPK and Baluchistan.

“*Yes, definitely cultural values don't change. In cities, like Mardan and Swat females have limited social life so they have literally no communication with males due to which they struggle to cope up with them if they have to work with males in future” (F.Pharm.07)*

“*Yes, one of the biggest cultural barrier is the language. Some males lack appropriate accent for communicating with females and are considered rude. Moreover, cultural favoritism during appointments is quite common, especially in KPK and Baluchistan and usually females are not preferred while hiring” (F.Doc.01)*

“*Yes it counts a lot as culture of one province vary from other. Mindset of people is dominated by their culture, however, it seems to be improved at higher posts. Unfortunately, in KPK still few families don't prefer working women and do not allow them to work” (M.Pharm.03)*

“*Yes, definitely it has an impact. We have examples of KPK and Baluchistan where it is very difficult for females to teach in universities. Although, situation is improving, but still there is long way to go” (M.Doc.04)*

#### Wearing a veil

Mixed responses were received on considering veil as a barrier toward promoting gender equity by female academicians. However, all the male academicians did not see veil as barrier toward gender equity.

“*No, it is not a big issue in academia. I think females covering their faces are seen with more respect in our country. It may varies according to the profession but I think mostly it is not an issue in academia” (F.Pharm.05)*

“*Yes, students are less comfortable with female teachers covering their faces as they can't see their expressions and may not pay proper attention towards them. Few male coworkers also hesitate to talk to such females” (F.Doc.02)*

“*No I don't think it is an issue in Pakistan and has anything to do with number of opportunities for females. It might be an issue in Europe or Western countries but not in Pakistan” (M.Pharm.07)*

“*I don't think there is any place in Pakistan where a female is rejected just because of her veil” (M.Doc.03)*

### Theme 4: Perceived traits, competency and commitment skill set among female healthcare academicians

Almost all the females as well as male healthcare academicians were of the view that female academicians are competent, hardworking and committed with their jobs.

“*I think both male and female academicians are equally competent but female academicians possess more assertiveness, better decision making, commitment and communication skillset” (F.Pharm.02)*

“*I think female academicians are more productive as they are more focused towards producing results in shorter duration. On the other hand, males are not so serious towards meeting tasks deadlines” (F.Doc.06)*

“*I feel females are more competent working in all fields including academia as compared*

*to males as they are more committed, convincing and dedicated” (M.Pharm.07)*

“*I think that female academicians are more punctual and committed. However, males are generally casual as they know the tactics to get the work done in short time span” (M.Doc.02)*

### Theme 5: Perceived teaching effectiveness among female healthcare academicians

Mixed responses were observed regarding teaching effectiveness of female academicians. Half of the females as well male healthcare academicians thought that female were more effective teachers while other half was of the view that teaching effectiveness is based on individual trait irrespective of gender.

“*I think female teachers possess the skills to make a hard subject easy for her students as they are more innovative than males” (F.Pharm.02)*

“*I think females are more hardworking than males and are far better teachers than them” (F.Doc.01)*

“*I believe it depends on an individual's personality and his/her ability to involve all the class. I think both genders can be equally effective” (F.Pharm.03)*

“*I think it is an individual trait and has nothing to do with gender. It all depends on competency of academicians as teaching is an art and only knowledge cannot improve it. It requires skills and competency for delivery” (M.Pharm.05)*

“*I had experience of being taught by both male and female teachers during my student life and based on my experience, I think that female teachers are more convincing and have better teaching effectiveness as compared to male teachers. I think reason for this is the element of softness in attitude, more commitment, better preparation of lectures and way of delivering the concept in the lecture” (M.Pharm.08)*

“*I believe females are very effective as far as teaching is concerned as they have better conceptual knowledge and delivery skills than males” (M.Doc.07)*

### Theme 6: Professional relationship of female healthcare academicians

Mixed responses were received by both male and female healthcare professionals toward professional relationship of female academicians with their counterparts and male colleagues.

“*Yes, I think female academicians support their female colleagues especially in private sector. I have seen such female role models academicians in private sector universities who support and train their female colleagues. It varies individual to individual as I have seen many males being mentors of their female colleagues and few giving them tough time” (F.Pharm.06)*

“*I think females are usually jealous and very keen observers. They never like other females to cross them. They don't generally work in harmony with each other. However, I have seen most of the male in a supporting role for their female coworkers. But it is difficult for them to except females as their boss” (F.Doc.07)*

“*I do not think that females support each other. However, I have witnessed many males supporting their female colleagues” (M.Pharm.02)*

“*Well, I think in my opinion jealousy factor exists among females, so in certain cases female academicians do not play supportive role for their colleagues, unless there is a strong friendship. But most of the males support their female colleagues” (M.Doc.01)*

### Theme 7: Representation of female healthcare academicians at leadership positions

Almost all the females as well as male healthcare academicians were of the view that female academicians are better administrators and possess leadership qualities but have less representation at leadership positions.

“*I believe females are very good administrators, if given the opportunity but they are usually not given fair chance to represent themselves at leadership positions” (F.Pharm.03)*

“*To be honest they do not have appropriate representation at leadership positions. However, they have the potential to lead the show more effectively” (F.Doc.02)*

“*I think although, trend is changing these days as more females are working at leadership positions and are playing their roles very well but still it has long way to go” (M.Pharm.01)*

“*I do not think females are given ample representation at leadership positions worldwide, especially in Pakistan” (M.Doc.02)*

### Theme 8: Research opportunities for female healthcare academicians

Almost all the females as well as male healthcare academicians were of the view that equal opportunities are available for both genders but still it seems difficult for female academicians to avail them due to their personal issues.

“*I think equal research opportunities are available for both genders but at times females could not avail them due to personal issues like field work or travelling” (F.Pharm.02)*

“*Equal opportunities exist for both genders but at times it gets difficult for females to avail them” (F.Doc.06)*

“*I think both have equal opportunities but at times it is difficult for females to do research along with their household duties” (M.Pharm.04)*

“*Both have equal opportunities but at times research is not a priority for them due to work life balance” (M.Doc.05)*

### Theme 9: Impact of academia as profession on married life

Almost all the females as well as male healthcare academicians were of the view that females being academician has an impact on married life.

#### Improved eligibility for marriage

Almost all the females as well as male healthcare academicians were of the view that being female academician improve eligibility for marriage.

“*I feel that it is an added advantage for marriage as most families prefer academicians as daughter in law due to the fixed job timing” (F.Pharm.06)*

“*Yes, I think most of the families prefer working women as academicians for marriage” (F.Doc.02)*

“*Yes, it has an added advantage for marriage as worldwide teaching is considered respectable job” (M.Pharm.08)*

“*Yes, it has benefit as working women are now preferred for marriage in Pakistan. Teaching is still recognized as a respectable profession and female academicians are obviously preferred” (M.Doc.08)*

#### Impact of higher salary than partner

Almost all the females as well as male healthcare academicians were of the view that having higher salary than the husband effect the marital life.

“*Yes, I think it is always difficult for the males to accept their partners being paid more than them and it effects the quality of their marital relationship” (F.Pharm.01)*

“*Yes it effects the marital life as the males in our society can't accept this fact and feel inferiority complex which leads to fights. Husbands always want to have that upper hand over their spouse in terms of financial literacy” (F.Doc.05)*

“*Yes, quality of marriage is effected by this as males suffer from inferiority complex” (M.Pharm.06)*

“*Yes it has an impact on the marriage. The impact might be positive as well as negative. Positive impact includes the addition of financial resources and sharing of burden. However, negative impact include jealousy and inferiority complex” (M.Doc.01)*

### Theme 10: Job turnover ratio among female healthcare academicians

Mixed responses were received from females as well as male healthcare academicians. Half of them were of the view that females switch more and the other half felt vice versa.

“*I think males switch jobs more than females as they have to support their families in terms of finances” (F.Pharm.02)*

“*In my view, females switch jobs more due to multiple reasons including out of station marriage, family restrictions, gender disparity and job workplace culture” (F.Doc.03)*

“*I think females generally prefer to work at the same place as they usually they get emotionally attached to workplace relatively more than males” (M.Pharm.05)*

“*I think males switch jobs more often than females to avail better opportunities in terms of finances to support their families” (M.Doc.02)*

### Theme 11: Strategies for improving gender equity disparities in academia of healthcare profession

All females as well as male healthcare academicians were of the view that by providing conducive environment, family & peer support, flexible working hours, transport and day care facilities can help to improve gender equity disparities for the female workforce.

“*I think the gender equity policies need to be devised and implemented to counter negative attitude of males as boss towards their competent female colleagues. However, I feel that only 10% of the females possess strong leadership qualities, therefore, we as females need to work on our leadership traits to address gender disparities at workplace” (F.Pharm.01)*

“*We have to provide the female workforce with favorable working environment. Day care facility should be provided for child bearing mothers. Transport issues must be resolved and females should get this facility for free” (F.Doc.06)*

“*I think a conducive working environment along with daycare and transport facilities can help to improve the situation. Moreover, family members especially males must encourage and empower them as working women” (M.Pharm.06)*

“*I think there should not be any discrimination in salaries of males and females having same position and qualification. Male academician in private sector are usually paid more than females. Moreover, transport facilities, flexible working hours, conducive work environment and most importantly open mindedness and supportive role of family members can help to promote gender equity” (M.Doc.01)*

## Discussion

Gender equity is still a challenge across various labor markets including health market. Physicians, nurses and pharmacists among many other professionals are no exception to the global trends of gender inequity at workplace. Gender equity is vital in healthcare profession in order to ensure effective, long term and sustainable career advancement for women. The results of the present study highlighted that majority of the female healthcare professionals were of the view that gender equity is not an issue in academia in Pakistan with the exception of females working in other fields and from underprivileged areas. However, majority of the male healthcare professionals perceived gender equity as an issue in Pakistan. Similar results were observed in a study conducted in Canada where gender inequity was observed in academia especially at leadership positions (16). As reported by Global Gender Gap Report 2014, Pakistan rank at 141 amongst the 142 countries in gender gap. The labor force participation was reported as 86 males and 25 females with female to male ratio as 0.30 (17). Despite the increased influx of women as healthcare professionals over the last decade, still horizontal and vertical occupational gender inequities exist. The results of the present study highlighted that majority of the female as well as male pharmacists were of the view that employment to population ratio of female academicians was low. On the other hand, male and female doctors were of the view that that employment to population ratio with respect to female doctors in academia was adequate. The findings are consistent with a study conducted in USA which concluded that male healthcare professionals were present in a higher employment ratio as compared to female healthcare professionals in academic healthcare centers (18). Women in healthcare fields face challenges related to poor work environments including limited opportunities for career advancement, work-related stress, unsatisfactory working conditions and unfavorable policies to promote leadership and salary disparities In addition, women in the US earned less than their male colleagues despite sharing equal amount of work, qualifications, experience and output. The current study revealed that non-conducive work environment, harassment at workplace and cultural issues were the main barriers identified toward gender equity in healthcare academia in Pakistan. Similar findings were reported in a study conducted in USA where cultural and workplace factors were highlighted as major barriers toward achieving gender equity (15).

Gender equity and women's empowerment have been set as prime goals by the United Nations for the 2030 global agenda for sustainable development. Although, progress has been made toward achieving these goals and women participation in the workforce has been growing rapidly over the course of time but still women occupy less than one third leadership and management positions. The current study reported that generally female academicians are perceived competent, hardworking and committed with their jobs. They are considered more effective teachers but have limited access to opportunities for leadership positions as compared to males. Similar results were reported by a study conducted in Germany where majority of the women doctors were of the view that they have less access to administrative positions as compared to their male counterparts (19). Gender inequity has been a major issue in developing countries. Pakistan has been facing this issue from generations where women are mostly deprived of their equal rights as compared to men. Most of the time they are not allowed to work or chose partner for marriage. The notion of working women are not well perceived in few provinces of Pakistan, especially the remote and tribal areas. The results of the study revealed that female academicians are generally more accepted in the society for marriage than any other profession. Generally, less support is provided by the male partners and usually most of them are dissatisfied with higher salary and career progression of their better halves. Similar results were reported from a study which showed that young professionals had poor marital relationships due to wage differences among partners (20).

Gender disparity is a worldwide phenomenon. This disparity is not only with respect to opportunities and resources but also in rewards, and exists in all regions and classes. Managerial positions are usually held by males, who not only have more decision making power but also have more opportunities of social networking. Women have to achieve a successful career at the cost of their family life. The results of the current study showed that equal research opportunities exist for both genders but female academicians are unable to access them due to their personal issues and family commitments. Almost, < 30% of the world's researchers are women (21). Inadequate support for women at the workplace result in work–life imbalance and affect family responsibilities, while stereotyping and inequity lead to stress, low life satisfaction and less productivity. Low life satisfaction affects overall performance of an individual both personally and professionally. Hence, implementing strategies like flexible timing and part-time work from home might improve motivation at the workplace, which in turn could strengthen and support gender equity across healthcare organizations. There is a critical need to develop organizational policies for promotion of gender equity through healthcare academia. The results of the current study highlighted that organizational policies should be introduced to promote work-life balance for women academicians. Conducive work environment, childcare support, transport facility, equal salary packages and family support could help to address the barriers linked to achieving gender equity in the country. These findings are in line with the results obtained from a study conducted in Canada where organizational support strategies helped in reducing gender disparity among academicians (5).

## Study limitation

The present study was conducted in twin cities of Pakistan and results may not be generalized to rest of the country. Responses of respondents may not depict their true feelings. Few of the respondents were reluctant for audio recording of the interview.

## Conclusion

The results of the present study concluded that majority of the male as well as female healthcare professionals perceived gender equity as an issue in academia in Pakistan, especially in underprivileged areas. Non-conducive work environment, harassment at workplace and cultural issues were the main barriers identified toward gender equity in healthcare academia in Pakistan. Equal research opportunities exist for both genders but female academicians are unable to access them due to their personal issues and family commitments. Generally, female academicians are perceived competent, hardworking, committed and more effective teachers but still have limited access to opportunities for leadership positions as compared to the males. Although, female academicians are generally more accepted in the society for marriage than any other profession but still less support is provided by the male partner. Appropriate organizational policies must be introduced to address recruitment and advancement processes to ensure quality between women and men; equal pay for equal work; recognition and rewards that are unbiased and based on contribution and performance; non-discriminatory approaches to care and family responsibilities; and genuine access to various positions and levels of leadership by removing gender-based barriers in order to improve status of gender equity and economic development in Pakistan.

## Data availability statement

The original contributions presented in the study are included in the article/supplementary material, further inquiries can be directed to the corresponding author.

## Ethics statement

The studies involving human participants were reviewed and approved by Hamdard University Islamabad Ethical Committee. The patients/participants provided their written informed consent to participate in this study. Written informed consent was obtained from the individual(s) for the publication of any potentially identifiable images or data included in this article.

## Author contributions

MM and AHu conceptualized the project and contributed equally to data analysis and interpretation. HS, HA, AHa, and AA contributed with data collection. MM, AHu, and AHa contributed with manuscript writing and revision. All authors read and approved the final manuscript.
